# Describing Characteristics and Differences of Neutrophils in Sepsis, Trauma, and Control Patients in Routinely Measured Hematology Data

**DOI:** 10.3390/biomedicines10030633

**Published:** 2022-03-09

**Authors:** Huibert-Jan Joosse, Albert Huisman, Wouter van Solinge, Falco Hietbrink, Imo Hoefer, Saskia Haitjema

**Affiliations:** 1 Central Diagnostic Laboratory, University Medical Center Utrecht, 3584 CX Utrecht, The Netherlands; h.j.joosse-3@umcutrecht.nl (H.-J.J.); a.huisman@umcutrecht.nl (A.H.); w.w.vansolinge@umcutrecht.nl (W.v.S.); i.hoefer@umcutrecht.nl (I.H.); 2 Department of Trauma Surgery, University Medical Center Utrecht, 3584 CX Utrecht, The Netherlands; f.hietbrink-4@umcutrecht.nl

**Keywords:** neutrophils, sepsis, trauma, bands, clustering

## Abstract

Neutrophils have an important role in the immune response. These cells can be subjected to an impaired function and a shift in population depending on disease states. In sepsis, this shift is recognized and flagged by automated hematology analyzers, including the presence of band neutrophils, while these cells, although present, appear not to be detected in trauma patients. To better understand this suspected error in flagging, we set out to distinguish neutrophil populations of these two patient groups and compared these with controls. Different data-driven methods were used compared to standard algorithms used by the software of the analyzers. Using K-means clustering, we extracted neutrophils from raw hematology analyzer datafiles, and compared characteristics of these clusters between the patient groups. We observed an increased neutrophil size for both sepsis and trauma patients, but trauma patients had a smaller increase. Trauma patients also had a high proportion of cells with relatively high nuclear segmentation, which is contradictory with the presence of band neutrophils. This, in combination with the smaller size increase, might explain the inability to flag band neutrophils in trauma.

## 1. Introduction

Neutrophils are among the body’s first cellular responders to infections and tissue damage. Their functionality and capability to limit pathogen invasion and remove pathogens (e.g., by phagocytosis) is essential in host defense. Impaired neutrophil function therefore leads to an impaired immune response and can result in exacerbation of infections towards sepsis [1]. The ability of neutrophils to take up pathogens by phagocytosis and their elimination are crucial in this context and depend on their level of maturity and composition, albeit that these two abilities are not necessarily directly linked [2,3].

After (severe) trauma, patients are known to be at increased risk for complications because of the activation of neutrophils after tissue damage through damage-associated molecular patterns (DAMPs) and microbe-associated molecular patterns (MAMPs) [4,5]. The activation and increase of neutrophils accompanied by a left-shift [6,7] and release of band neutrophils [8] after trauma—from here on referred to as “trauma bands” vs. “sepsis-bands”—has been linked to increased risk for infection and, eventually, organ failure [4,5,6,9,10]. Although microscopically indistinguishable from the band neutrophils released during sepsis, their counterparts in trauma patients are not detected as such by the current cell identification algorithms used in automated hematology analyzers [8].

Current hematology analyzers for automated five-parameter differential counts—i.e., whole blood cell counts differentiating the following five leukocyte populations: monocytes, lymphocytes, neutrophils, eosinophils, and basophils [11]—are equipped to identify band neutrophils and issue a warning. Considering the susceptibility of (severe) trauma patients to adverse events such as infection and organ failure, the recognition of immature (band) neutrophils (versus mature ‘segmented’ neutrophils) is essential in order to make an early identification and prevent deterioration of the health of patients after trauma.

In the current study, we investigated why “trauma bands” are not flagged by our analyzers and what makes them differ from normal segmented neutrophils and “sepsis bands” by data-driven clustering methods based on raw measurement data.

## 2. Material and Methods

### 2.1. Population & Data

We selected hematology data as measured by the Abbott Cell-Dyn Sapphire hematology analyzer (Abbott Diagnostics, Santa Clara, CA, USA). Data from 20 well-phenotyped sepsis and trauma samples from previous collaborations were kindly provided by the traumatology department and consisted of surgical infection patients (‘sepsis’) and multitrauma patients (‘trauma’) as described before [8]. Patients were measured during their stay in the Intensive Care Unit (ICU), where band neutrophils were observed during microscopic examination of a routine blood smear. Trauma patient samples (*n* = 4) were of multitrauma patients that were hemodynamically stable, all taken on the second day after trauma. Sepsis samples (*n* = 7, of which 6 were intra-abdominal sepsis, 1 mediastinitis) were of sepsis patients that suffered from sepsis after surgery, all taken on the first day of sepsis. Samples without band neutrophils were selected as controls (*n* = 9).

Venous blood sampling was performed via venipuncture using 2.0 mL K_2_EDTA blood collection tubes (BD Vacutainer, Becton Dickinson, NJ, USA). Samples were kept at room temperature and were measured within 2 h after venipuncture in the ISO15189 certified Central Diagnostic Laboratory of the UMC Utrecht on a Cell-Dyn Sapphire. The reliability and validity of the laboratory results are monitored through routine quality control.

For each of these patient samples, hematology data were extracted from the Utrecht Patient-Oriented Database (UPOD). UPOD is an infrastructure of relational databases comprising data on patient characteristics, hospital discharge diagnoses, medical procedures, medication orders, and laboratory tests for all patients treated at the University Medical Center Utrecht (UMC Utrecht), Utrecht, the Netherlands, since 2004. The structure and content of UPOD have been described in more detail elsewhere [12]. UPOD has collected over two million hematology analyzer measurements including the underlying raw data files (FCS-files). FCS-files are standardized files for flow cytometry data [13]. For the white blood cells, five different variables (i.e., channels) are reported by the Abbott Cell-Dyn Sapphire [6,14,15]: the axial light loss (ALL), intermediate angle scatter (IAS), polarized and depolarized side scatter (respectively PSS and DSS), and the fluorescence (FL3; 630 ± 30 nm).

ALL is used as a measure of cell size, whereas the IAS is a measure of the cytoplasmatic complexity (granulation) of a cell. PSS is linked to the level of nuclear segmentation, and DSS is a measure of depolarization. Lastly, FL3 is a measure of cell viability (measurement of the uptake of the fluorescent dye propidium-iodine by non-viable cells).

### 2.2. Data Analysis

We extracted data on the five measurements (ALL, IAS, PSS, DSS, FL3) from the FCS files for each patient and combined them into one analysis file. We standardized the data so that for each variable the mean was 0 and the standard deviation was 1. In order to find leukocytes that were comparable with each other, we clustered the data using K-means clustering [16], and used the silhouette score and K-means inertia score to find the optimal number of clusters for these data. K-means clustering arbitrarily assigns data to k clusters, and iteratively updates the cluster centers, assigning data to their closest centers, until these centers remain stable. Ultimately, the data should be assigned to clusters that each contain distinct subsets of the data. The silhouette score defines how well-separated the clusters are, whereas the K-means inertia score is a result of the K-means algorithm, and is a measure of how well the algorithm was able to minimize the sum of squared distance of samples, so that samples are closest to their allocated cluster center [16].

After modeling the optimal number of clusters, we calculated the proportion of cells that belonged to a patient for each cluster, and then combined these proportions according to patient groups in order to calculate the distribution of these proportions per cluster and group. Additionally, we selected the clusters that corresponded to neutrophils, pooled these data, and then retrieved the distribution for the variables for each subgroup. The means for the groups per variable were compared using an analysis of variance (ANOVA) for normally distributed variables, and a Kruskal–Wallis test for non-normally distributed variables. If differences were found, we performed post hoc analyses using a T-test for normally distributed data or a Wilcoxon Rank Sum Test if the data were non-normally distributed. We tested normality using the Kolmogorov–Smirnov test.

We used the Python programming language to perform the analyses (version 3.7). For the K-means clustering the Scikit-learn (version 0.24) package was used, and for the statistical analyses the SciPy (version 1.6) package was used.

## 3. Results

In total, data from 20 samples were selected for this study, of which 7 samples were from sepsis patients, 4 samples were from trauma patients, and 9 samples were of control patients. For each of the sepsis and trauma patient samples, the presence of bands had been confirmed using microscopic examination of the peripheral blood smear. For all patients, an average of 6603 leukocytes were measured (range 2069–15,109). For controls, this number was 4189 (2069–5570), for sepsis patients the number was 9728 (5451–15,109), and for trauma patients it was 6576 (4404–11,896). The mean (SD) values for each of the studied FCS characteristics are given in Table 1. Here we found some notable differences, for example a larger cell size (ALL) for trauma (18,622 vs. 17,044 (controls), *p* < 0.001) and sepsis patients (20,833 vs. 17,044 (controls), *p* < 0.001), and an increase of FL3 signal in sepsis (20,833 vs. 17,044 (control) and 18,622 (trauma), *p* < 0.001). The differences that were found were observed across the whole leukocyte population, which also included lymphocytes and monocytes. As we were interested in the neutrophil population, we continued to further scrutinize the data using K-means clustering, which was able to distinguish the neutrophil population in our data.

### 3.1. Results of K-Means Clusters

**Figure 1:**
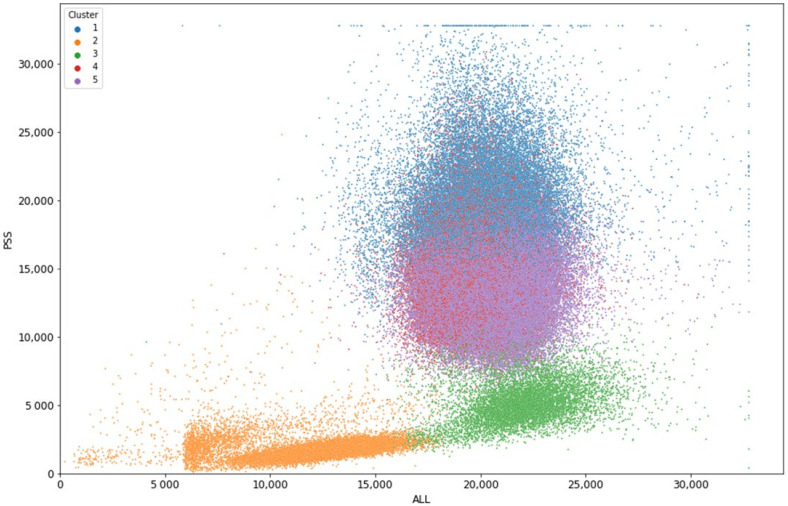
ALL versus PSS, colorized according to the K-means clustering with 5 clusters. Cluster 1 (blue), 4 (red), and 5 (violet) are corresponding to different populations of neutrophils. Clusters 2 (orange) and 3 (green) describe lymphocytes and monocytes, respectively.

Based on the silhouette score, we clustered the data into five clusters using K-means clustering, see Figure S1 for further explanation. In Figure 1, the ALL and the PSS measurements of the cells are shown, colorized according to the cluster they were assigned to using the K-means clustering. Cluster 1, 4, and 5 describe similar cells, namely neutrophils. Clusters 2 and 3 are lymphocytes and monocytes, respectively. The descriptive statistics for each of the clusters are given in Table 2.

**Figure 2:**
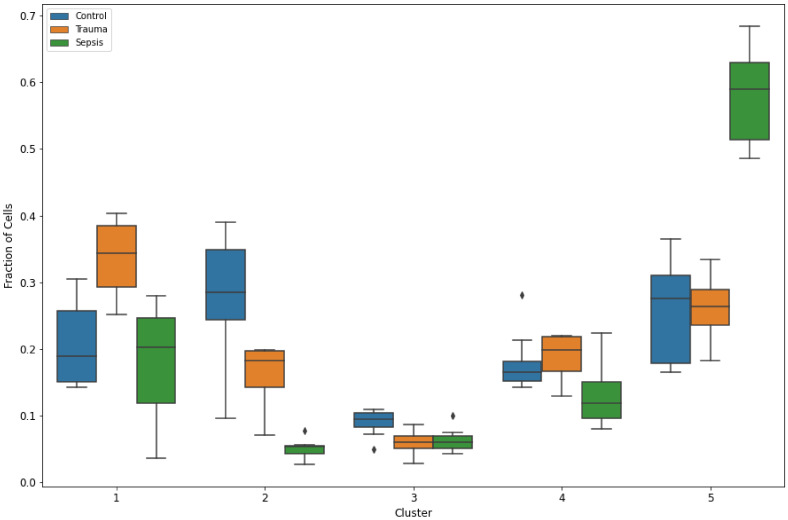
Fraction of cells in the different clusters for each patient group. In cluster 1, a relatively high proportion of cells of trauma patients were found, in cluster 2 and 3 this was true for control patients. In cluster 5, a high proportion of cells of sepsis patients were found. Clusters 1, 4, and 5 correspond to neutrophils, cluster 2 corresponds to lymphocytes, cluster 3 to monocytes. The black diamonds annotate outlier values (>1.5 interquartile range).

To further disentangle what characterizes these distinct clusters, we calculated the fraction of cells measured in each cluster per patient, and, subsequently, the patients were grouped according to their condition (trauma, sepsis, or control). Figure 2 shows the distribution of each cluster for each disease state. In cluster 1 we observed a relatively high proportion of cells for trauma patients (*p* < 0.05 vs. sepsis, *p* < 0.005 vs. controls). In cluster 5 we observed a relatively high proportion of cells for sepsis patients (*p* < 0.001 vs. trauma and controls). In cluster 2 (*p* < 0.05 vs. trauma, *p* < 0.001 vs. sepsis) and 3 (*p* < 0.05 vs. trauma and sepsis) we found a higher proportion of cells for the controls, which also implied that controls had fewer neutrophils.

### 3.2. Selecting the Neutrophils

**Figure 3:**
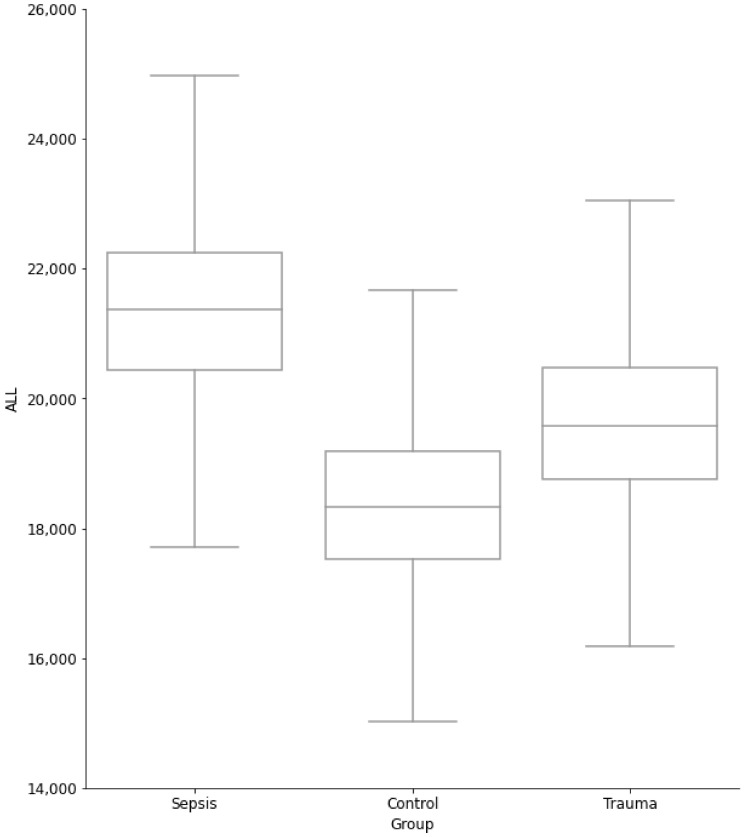
Boxplots for the three groups for the ALL measurement after combining all clusters that described a subpopulation of neutrophils. Sepsis patients had, on average, the largest neutrophils (as measured by ALL), followed by trauma patients. The controls had, on average, the smallest number of neutrophils.

After selection and pooling of neutrophil clusters (1, 4, and 5) per patient group, we found a distinct and significant difference (H-statistic = 6547.69, *p* < 0.001) between all the groups and for all comparisons between groups, for the ALL channel, which roughly corresponded with the size of a cell (Figure 3 and Table S1). Boxplots for all other channels can be found in Figures S2–S5.

### 3.3. Different Clusters within Neutrophils

**Figure 4:**
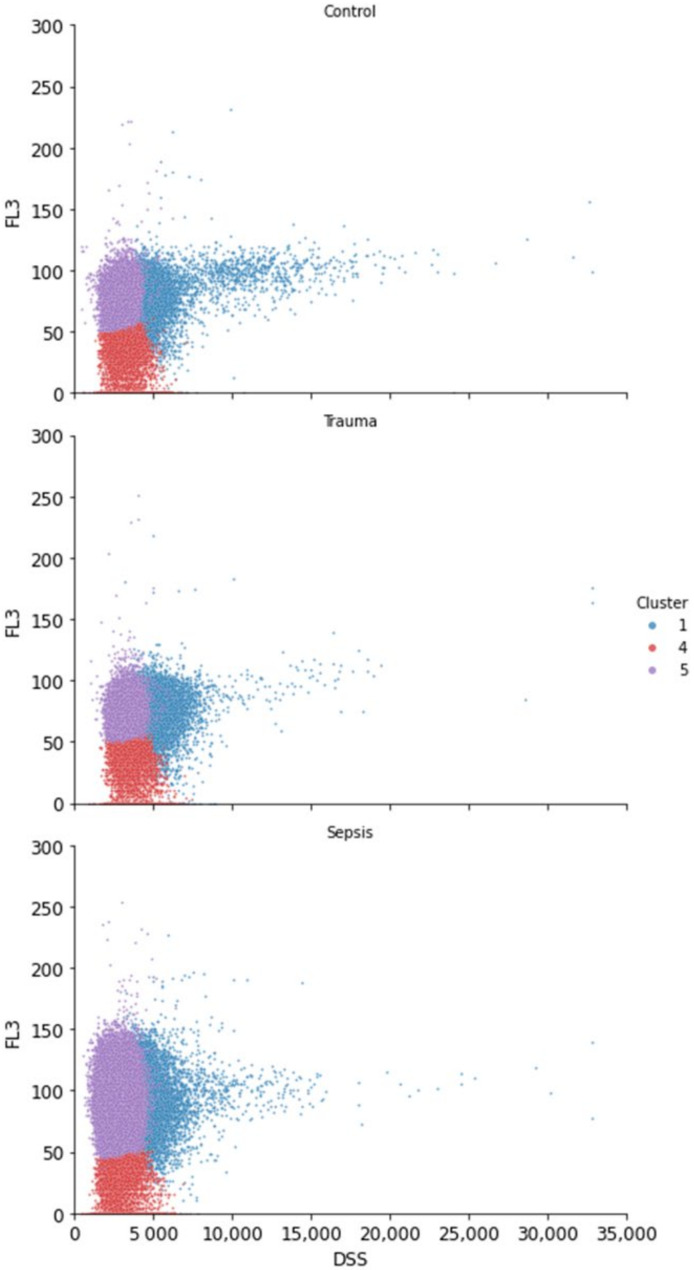
What defines the three neutrophil clusters? Cluster 1 (blue) is described by relatively high DSS/PSS and FL3 values, cluster 4 (red) is described by low FL3 and DSS/PSS values, and cluster 5 (violet) is described by relatively high FL3 values and low DSS/PSS values.

While the combination of clusters 1, 4, and 5 encompassed the whole neutrophil population, these three clusters still had distinct characteristics, thus describing distinct groups of neutrophils. After scrutinizing the data for the distinction between the three neutrophil clusters, we found the distinction of these clusters in the combination of the DSS/PSS and FL3 variables. Relatively high FL3 and DSS/PSS measurements were seen for cells in cluster 1, while the opposite (low FL3 and DSS/PSS) was true for cluster 4 (Figure 4 and Figure S6). Figures S7–S11 show the univariate distributions per cluster for the five variables. Cluster 5 was defined by the relatively high measurement of FL3 and a relatively low measurement of DSS/PSS. Combining the information from Figure 2 with these findings, the trauma patients had a higher proportion of cells with higher DSS/PSS and FL3, while the sepsis patients had a higher proportion of cells with lower DSS/PSS and high FL3.

## 4. Discussion

In this data-driven hypothesis-generating study, we investigated why automated hematology analyzers are unable to identify neutrophil bands in patients with trauma, using data on leukocytes from three patient groups: sepsis, trauma, and controls. We found most noticeable distinguishing features related to neutrophils in these groups which might help explain why analyzers are unable to flag trauma neutrophil bands.

As expected, controls showed a lower proportion of neutrophils and a higher proportion of monocytes and lymphocytes. Our analysis indicated that neutrophil size was largest in sepsis patients and smallest in controls, as derived from axial light loss (ALL). Interestingly, sepsis patients tended to have a higher measure for FL3, an inverse measure for cell viability, and lower nuclear segmentation (i.e., PSS/DSS). When describing trauma patients, we found that these had a relatively high number of cells with increased FL3 intensity, but this did not result in a noticeable shift in FL3 compared to controls. In contrast to the neutrophils from sepsis patients, these cells had a relatively high level of segmentation, as measured by the DSS/PSS channel.

Taking a closer look at what might explain the inability of analyzers to determine a classification of a neutrophil subtype (i.e., segmented neutrophil versus band neutrophil) we found two possible explanations. First of all, our data showed an increase in ALL for sepsis and trauma patients, which was in line with earlier findings where the increase of neutrophil size was seen in sepsis [17,18], and increased neutrophil size was also observed for trauma patients, along with an increase in cell complexity [6]. Yet the increase in neutrophil size for trauma patients was less, compared to sepsis patients. At the same time, trauma bands were not larger compared to segmented neutrophils in trauma as all cells increased size in this population [8], which might explain the inability of flagging trauma bands.

Secondly, by DSS/PSS measurements, there was a proportional increase of segmented cells in trauma cells. This overall increase contradicted the presence of band neutrophils and might even wipe out any population-effect of bands on the measurement of segmentation (a lower DSS/PSS). This might cause analyzers not to recognize the presence of bands. Considering the confidentiality of the algorithm in the Cell-Dyn Sapphire analyzers, and the hypothesis-generating nature of this research, the role and impact of each of these variables on band flagging remains elusive and should be the focus for further research. Automated hematology analyzers are able to flag samples based on the presence of band neutrophils yet are not able to directly measure these cells. This complicates defining the characteristics of these cells in automated hematology analysis, as there are heterogeneous characteristics, e.g., size differences of band neutrophils in different patient populations [8].

With regards to the increase in the non-viability measure in our data (FL3) we observed an increase for sepsis patients, which was not seen in trauma patients. To interpret these data adequately, it is important to note that the FL3 channel did not show a clear separation of viable and non-viable cells. The cut-off for cells being classified as non-viable by the intensity of FL3 fluorescence was based on evidence from millions of measurements and serves its purpose for clinical application, e.g., separation of fresh blood samples from older samples [19,20]. However, it should be noted that the reagent, propidium-iodide (PI), does not bind exclusively to DNA, but also to RNA [11]. In real-life practice, this does not matter as the fact that PI crosses the cell membrane suffices to estimate cell viability. As mentioned above, however, our data did not show a clear separation of cells. Unfortunately, it is impossible to differentiate between cytoplasmatic FL3 signal which might be indicative of RNA binding or nuclear staining, reflecting binding to nuclear DNA. Whether a cell was indeed apoptotic/necrotic once FL3 signals exceed the reference cut-off at around 100AU remained unclear in our patient populations. For example, viable neutrophils from critically-ill patients exerted high fluorescence as a result of in vitro manipulation, such as red blood cell lysis in automated hematology analysis. Additionally, extracellular DNA and autofluorescence also influenced the measure of FL3 [19]. Considering this, the increase of FL3 in sepsis patients could be an increase of non-viable cells but could also be an effect of increased amounts of extracellular nucleic acids (i.e., DNA and RNA).

If trauma patients did have a more viable neutrophil population, a possible explanation could be that the underlying trigger might be less harmful to neutrophils than the invading pathogens causing (mostly bacterial) sepsis. As a consequence, this might result in relatively more ‘non-viable’, fragile neutrophils in sepsis patients, resulting in higher FL3 measurements.

Lastly, these differences in the neutrophil characteristics were not the only differences found between the groups. We also observed an increased proportion of neutrophils in trauma patients and sepsis patients which could be expected, considering the activation and mobilization of neutrophils in these pathological states, thus increasing the proportion of neutrophils.

This study was limited in size, and performed using one type of hematology analyzer, which was why we cannot provide a definitive answer as to what these three subpopulations characterize. We, however, saw a consistent pattern between these patient groups in our data. More research in larger patient populations is needed to provide more insight into the clinical value of combining ALL with FL3 and DSS/PSS to improve the identification of band neutrophils in trauma patients.

In conclusion, based on our data-driven hypothesis-generating approach on routine hematology data, we were able to identify differences in neutrophil characteristics in sepsis, trauma, and control patients which might explain why analyzers are unable to automatically recognize the presence of band neutrophils in trauma patients based on current algorithms. Aside from a smaller increase of cell size in trauma patients compared to sepsis patients, we observed that trauma patients had an increased proportional count of cells with high average side scatter measurements, pointing to increased segmentation of neutrophils in these patients, contradicting the presence of a subset of band neutrophils, and possibly neutralizing the effect these bands had on the population effect on segmentation. These two findings might explain the inability to recognize and flag these samples by automated hematology analyzers. Finally, we also observed an increase in neutrophils with high FL3 values in sepsis patients, a measure for non-viability of cells.

## Figures and Tables

**Table 1:** Means (SD) of hematology characteristics, age, and sex.

Variable	IAS (AU)	ALL (AU)	PSS (AU)	DSS (AU)	FL3 (AU)	Age (Years)	Sex, Female (%)
Overall (SD)	16,677 (3484)	19,303 (3605)	13,084 (6236)	2879 (1827)	67.1 (35.0)	55.1 (17.5)	52.4
Control (SD)	15,307 (3985)	17,044 (3531)	11,362 (7055)	2559 (2241)	55.5 (34.5)	49.8 (20.3)	60.0
Sepsis (SD)	17,689 (2955)	20,883 (2945)	13,390 (4975)	2830 (1431)	77.3 (34.2)	66.1 (8.3)	57.1
Trauma (SD)	16,152 (3216)	18,622 (3329)	14,426 (7092)	3348 (1933)	59.4 (31.0)	49.0 (15.7)	25.0

**Table 2:** Mean (SD) (AU) for each variable and predominant cell type in each cluster.

Variable	Cluster 1	Cluster 2	Cluster 3	Cluster 4	Cluster 5
IAS	18,971 (2076)	9540 (1657)	13,590 (1915)	17,565 (1554)	17,828 (1602)
ALL	20,155 (2274)	11,760 (2702)	21,895 (2214)	19,717 (1740)	20,585 (1807)
PSS	20,005 (3337)	1790 (1030)	5467 (1789)	14,872 (3346)	13,480 (2464)
DSS	5022 (1728)	170 (256)	639 (436)	3209 (903)	2823 (664)
FL3	82 (20.5)	41.2 (39.3)	56.7 (30.4)	19.6 (20.4)	86.3 (19.3)
Cell type	Neutrophil	Lymphocyte	Monocyte	Neutrophil	Neutrophil

## Data Availability

The data presented in this study are available upon reasonable request from the corresponding author. The data are not publicly available due to privacy issues.

## Supplementary Materials

The following supporting information can be downloaded at: https://www.mdpi.com/article/10.3390/biomedicines10030633/s1. Figure S1: Scores with a different number of clusters for the K-means alghoritim, Figure S2: Distributions for the Polarized Side Scatter (PSS) variable, Figure S3: Distributions for the Depolarized Side Scatter (DSS) variable, Figure S4: Distributions for the Intermediate Angle Scatter (IAS) variable, Figure S5: Distributions for the fluorescence variable (FL3; 630 ± 30 nm), Figure S6: Scatterplot of the neutrophil data, with the Polarized Side Scatter (PSS) and Fluorescence (FL3; 630 ± 30 nm) plotted against each other, colorized according the clusters assigned by the K-means clustering, Figure S7: Density plots of FL3 distributions, colorized according to cluster from K-means clustering, Figure S8: Density plots of PSS distributions, colorized according to cluster from K-means clustering, Figure S9: Density plots of DSS distributions, colorized according to cluster from K-means clustering, Figure S10: Density plots of IAS distributions, colorized according to cluster from K-means clustering, Figure S11: Density plots of ALL distributions, colorized according to cluster from K-means clustering.
