# Supplementary material for: Describing Characteristics and Differences of Neutrophils in Sepsis, Trauma, and Control Patients in Routinely Measured Hematology Data
Source: Biomedicines. 2022 Mar 9;10(3):633. doi: 10.3390/biomedicines10030633 (PMC8945568; doi:10.3390/biomedicines10030633)
Supplement: Supplementary file 1 [file biomedicines-10-00633-s001.zip › Supplementary Figures.pdf]

Supplementary Figures

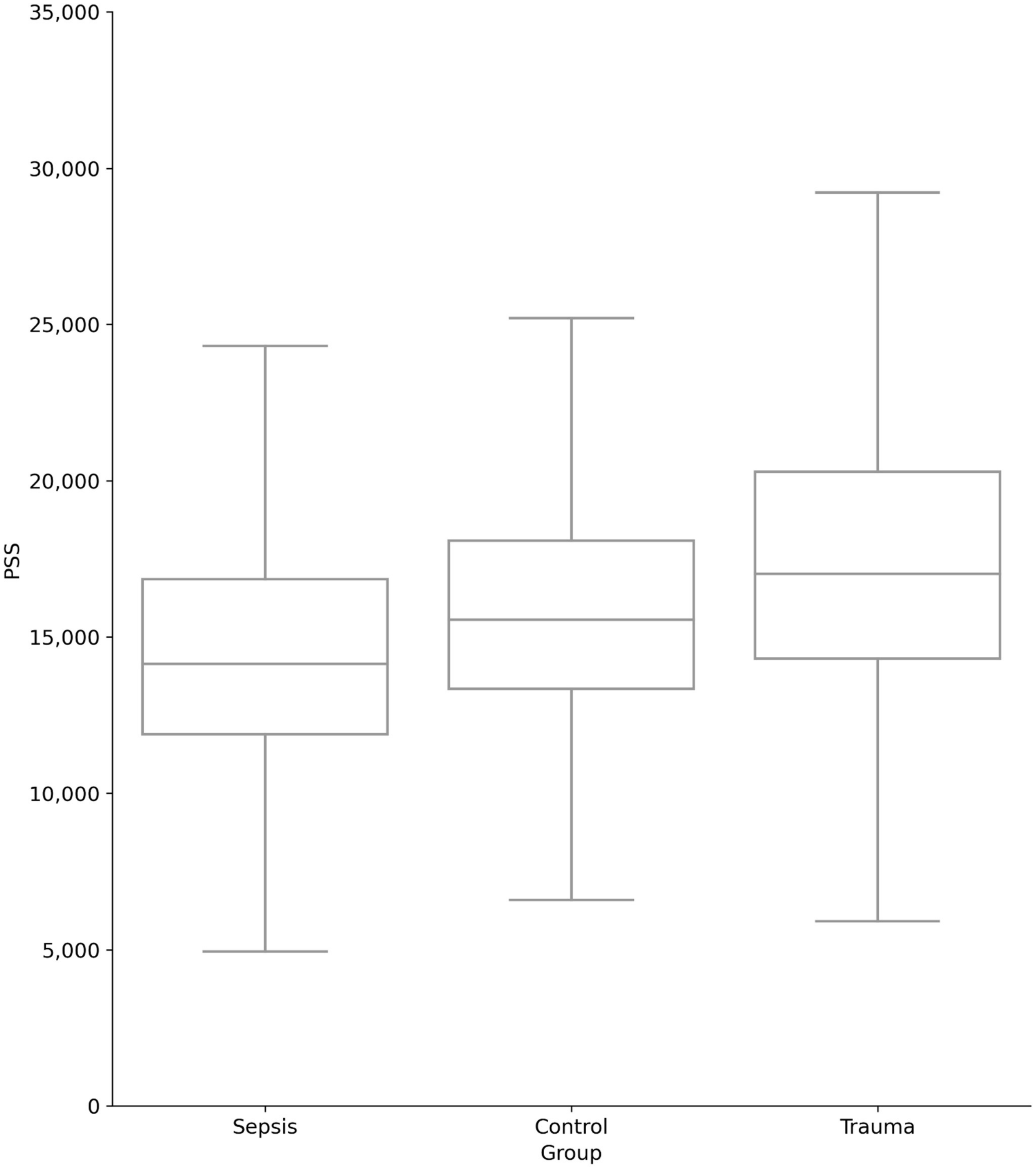

Figure S2. Distributions for the Polarized Side Scatter (PSS) variable. An increase is seen for the trauma neutrophils with regards to controls, a decrease for sepsis neutrophils.

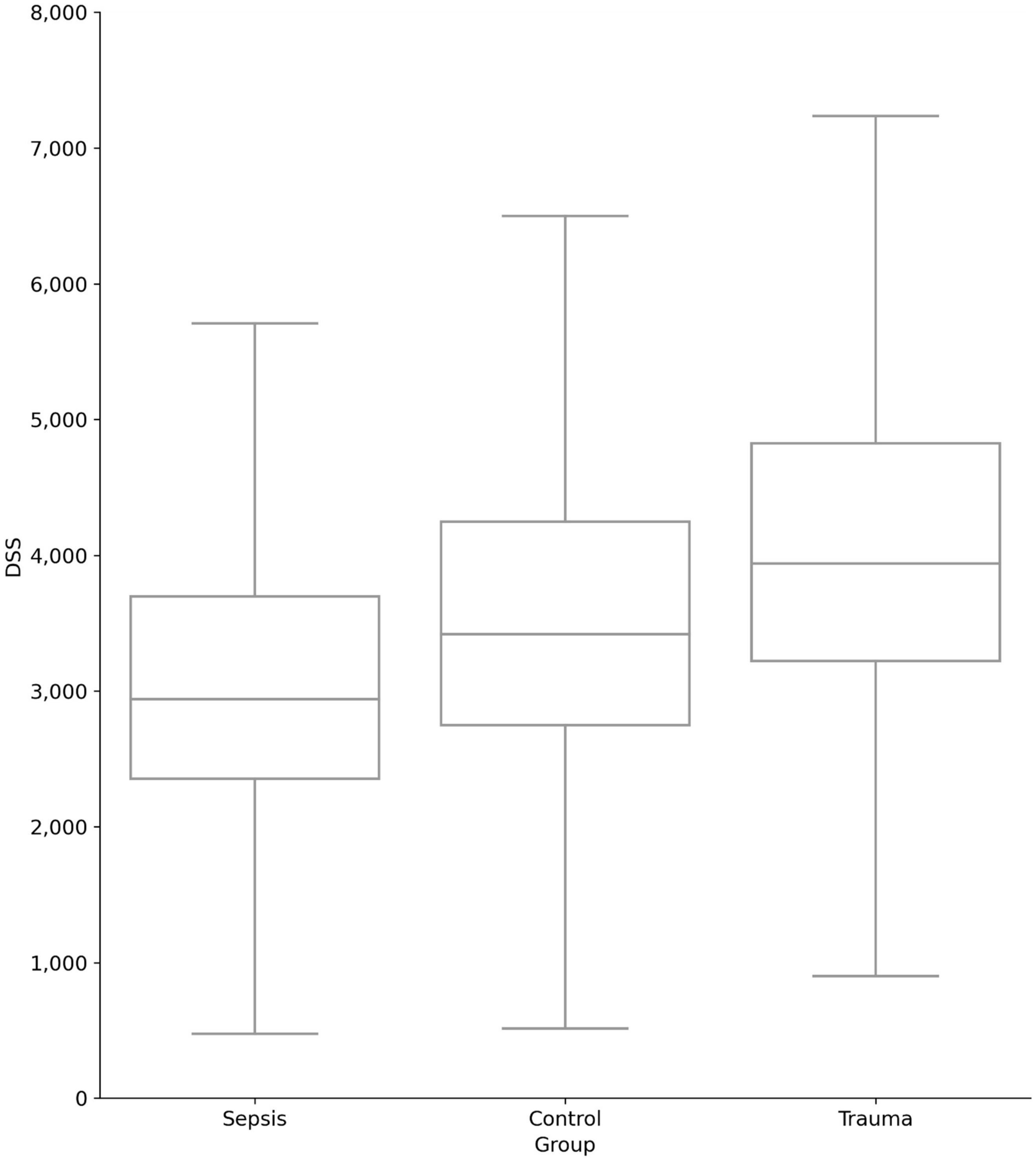

Figure S3. Distributions for the Depolarized Side Scatter (DSS) variable. As with the PSS variable, an increase is seen for the trauma neutrophils with regards to controls, a decrease for sepsis neutrophils.

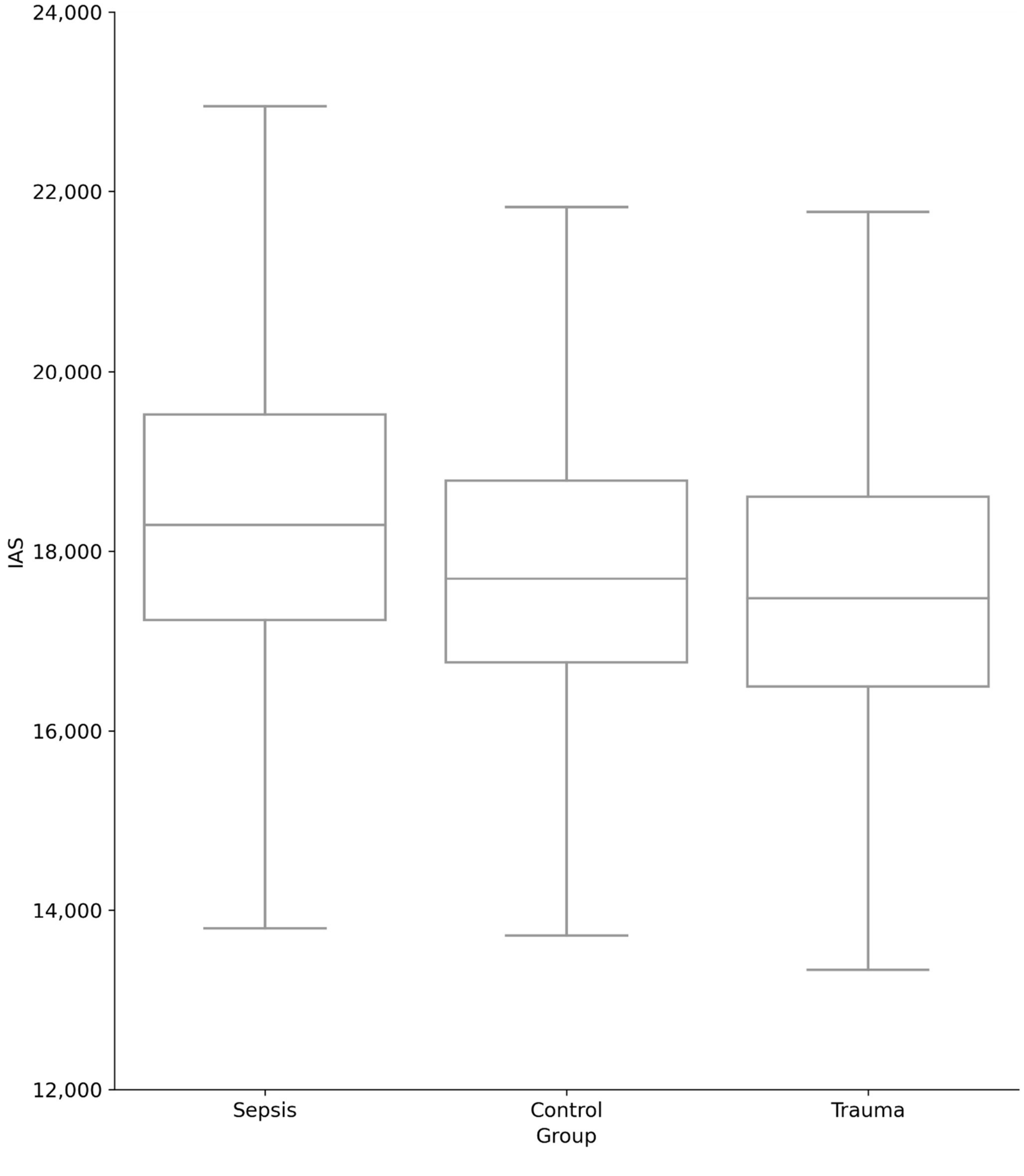

Figure S4. Distributions for the Intermediate Angle Scatter (IAS) variable. No large differences are found, neutrophils in sepsis seem to have a marginally larger measure for this variable.

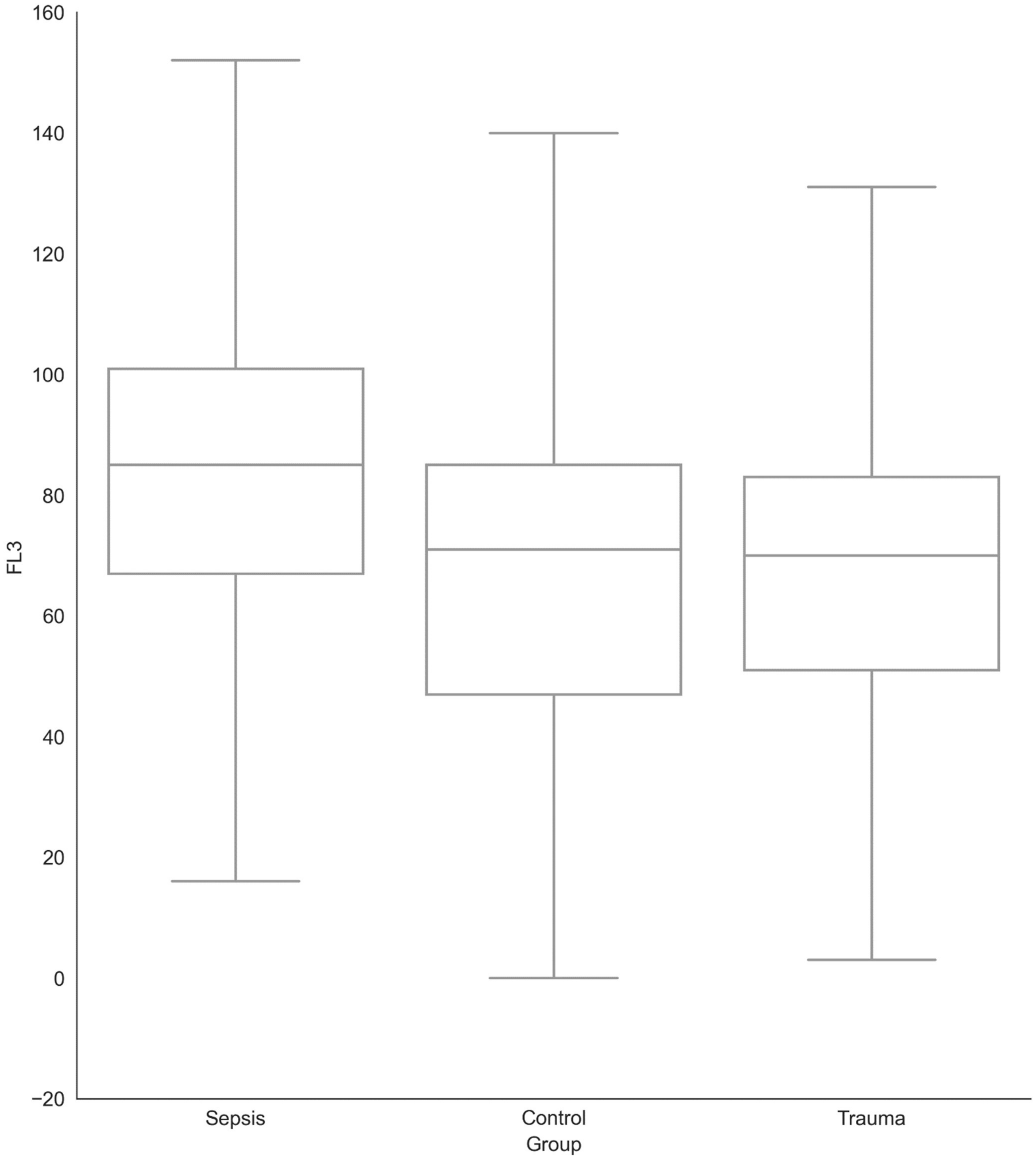

Figure S5. Distributions for the fluorescence variable (FL3; 630±30 nm). An limited increase is seen for the sepsis neutrophils with regards to controls and trauma neutrophils. The distribution for neutrophils in trauma and control patients are similar.

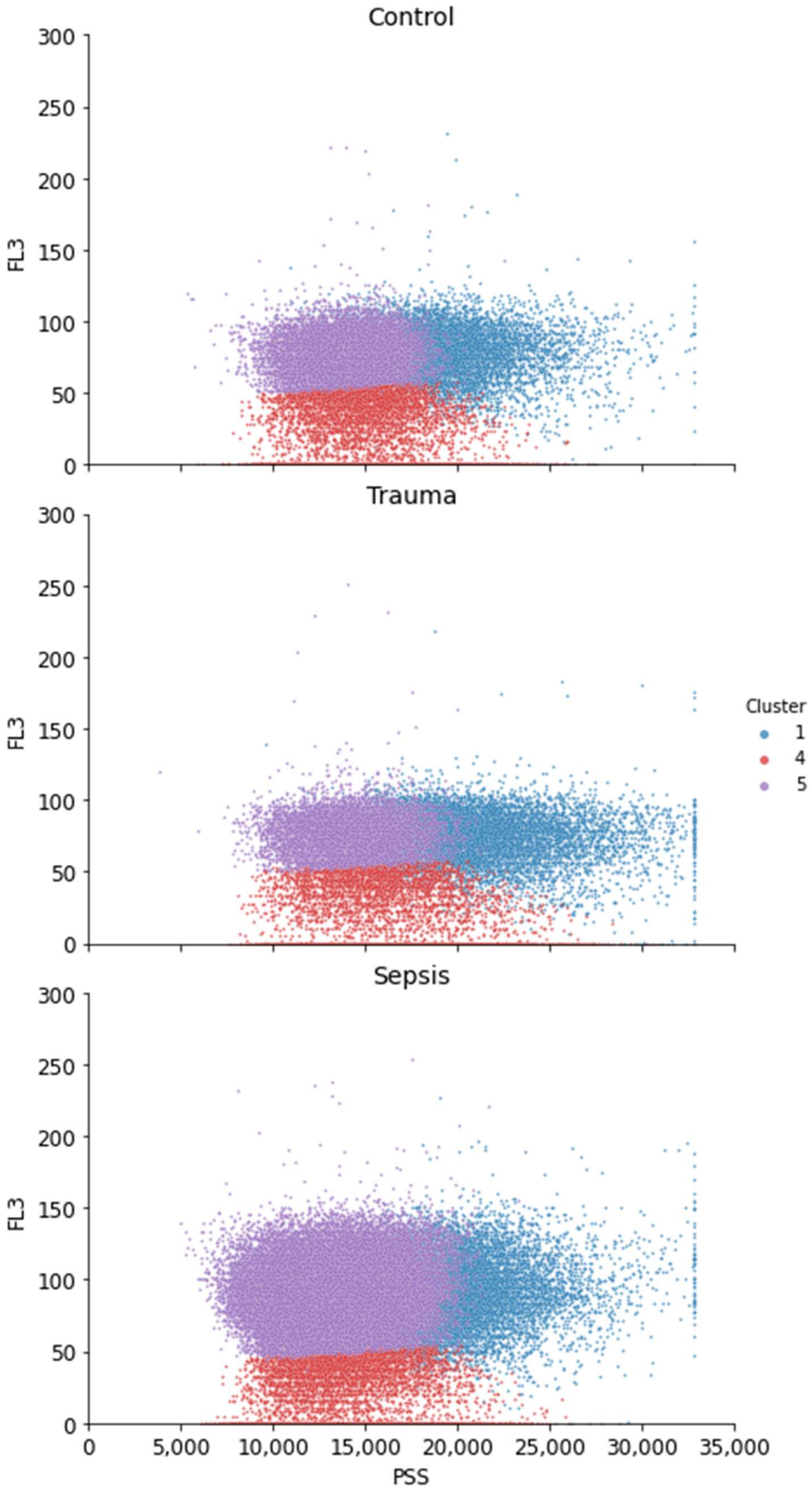

Figure S6. Scatterplot of the neutrophil data, with the Polarized Side Scatter (PSS) and Fluorescence (FL3; 630±30 nm) plotted against each other, colorized according the clusters assigned by the K-means clustering.

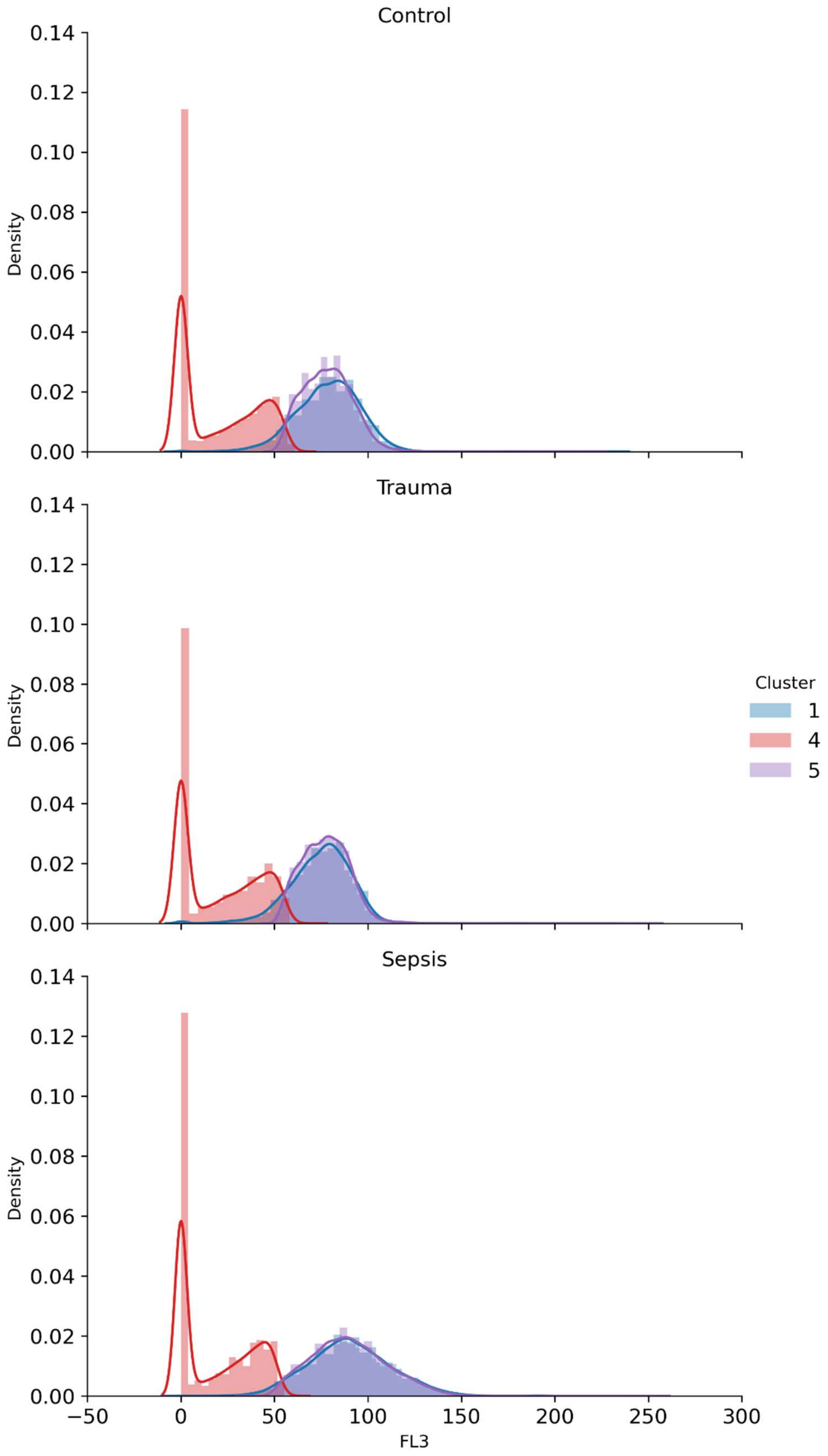

Figure S7. Density plots of FL3 distributions, colorized according to cluster from K-means clustering. Cluster 4 has a distinct distribution in contrast to cluster 1 and 5, as it contains lower values for FL3.

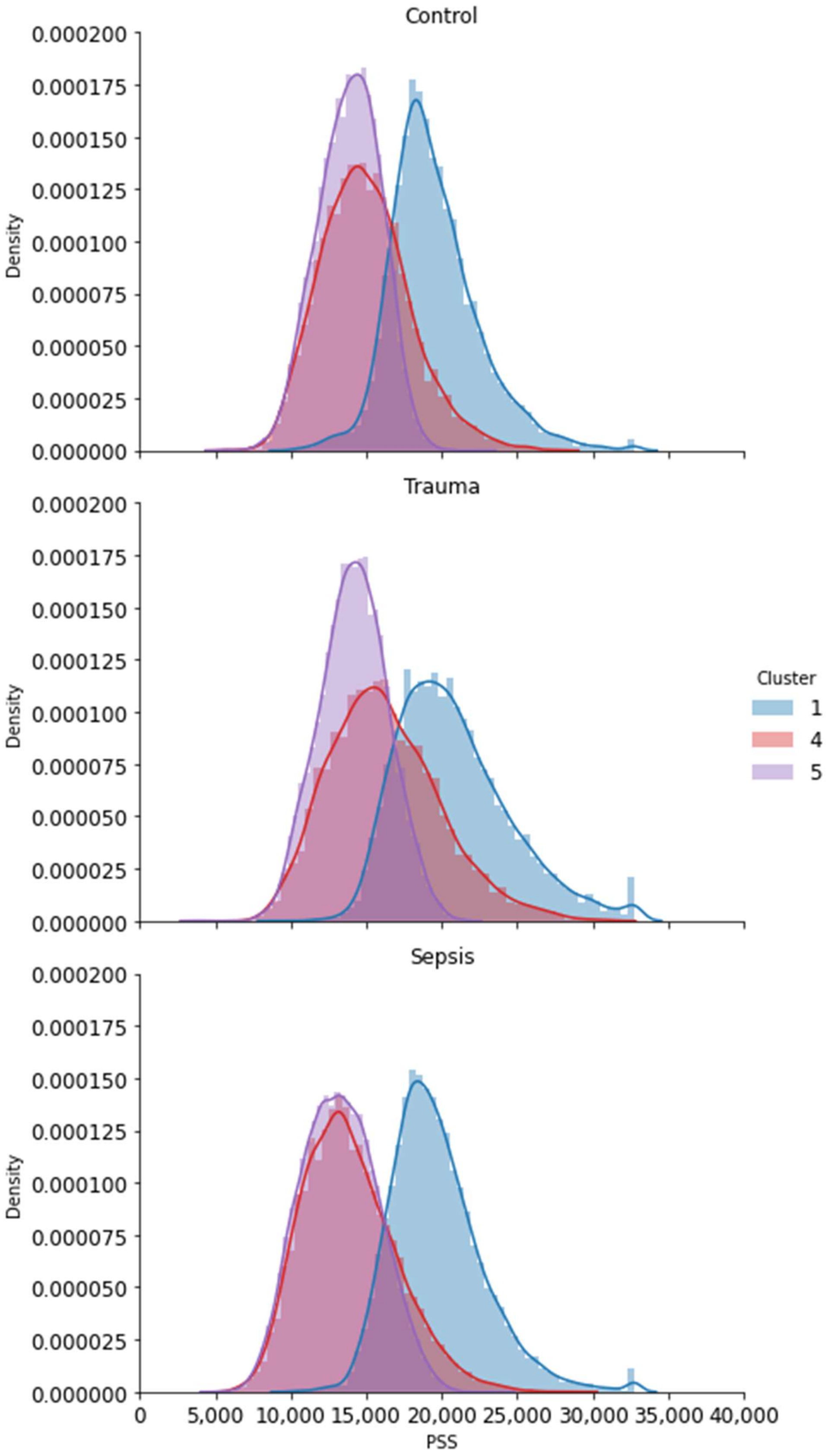

Figure S8. Density plots of PSS distributions, colorized according to cluster from K-means clustering. The distribution of cluster 1 is different compared to clusters 4 and 5, as it contains higher values for PSS.

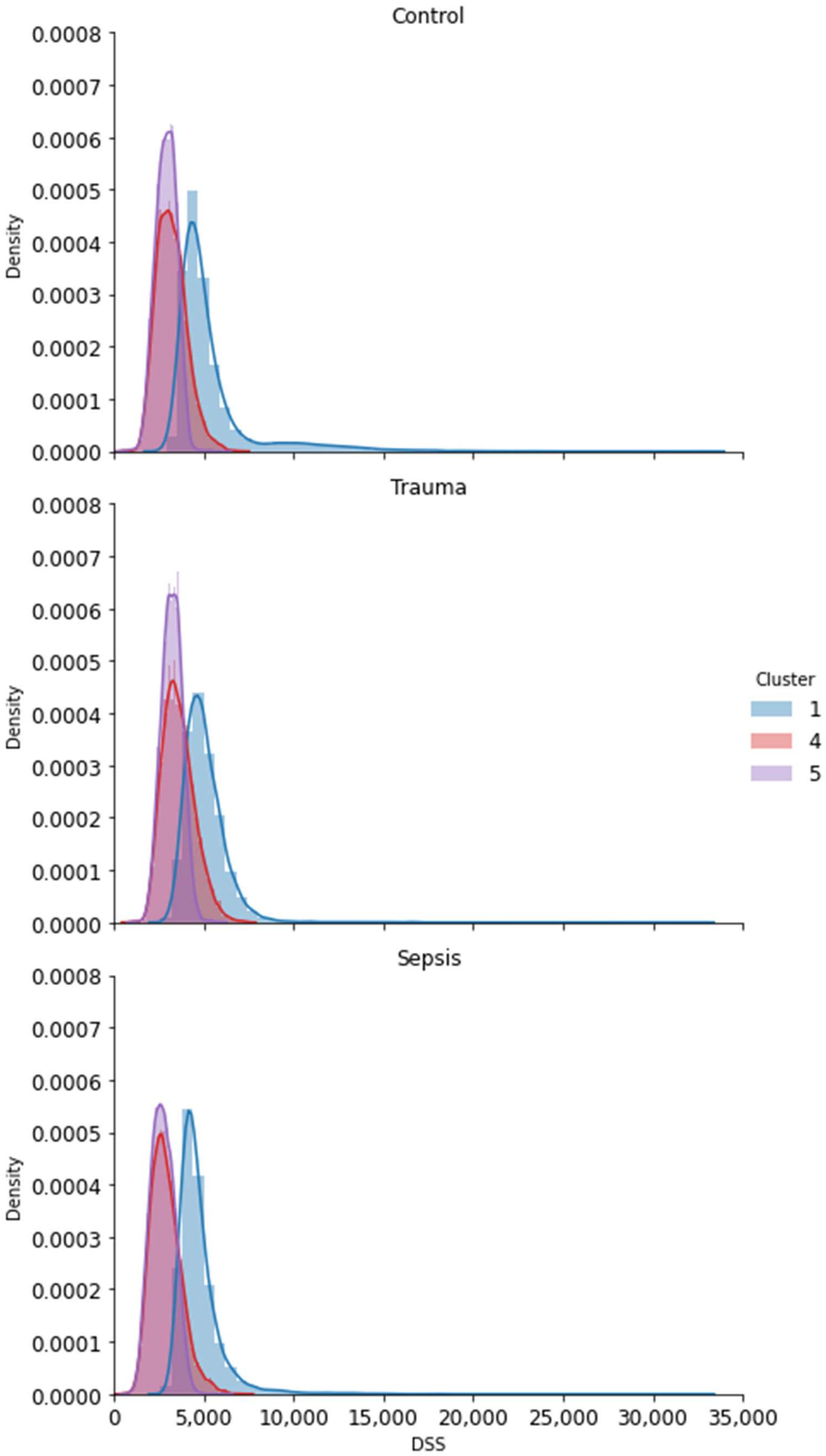

Figure S9. Density plots of DSS distributions, colorized according to cluster from K-means clustering. Comparable to PSS, the distribution of cluster 1 is different compared to clusters 4 and 5, as it contains higher values for DSS.

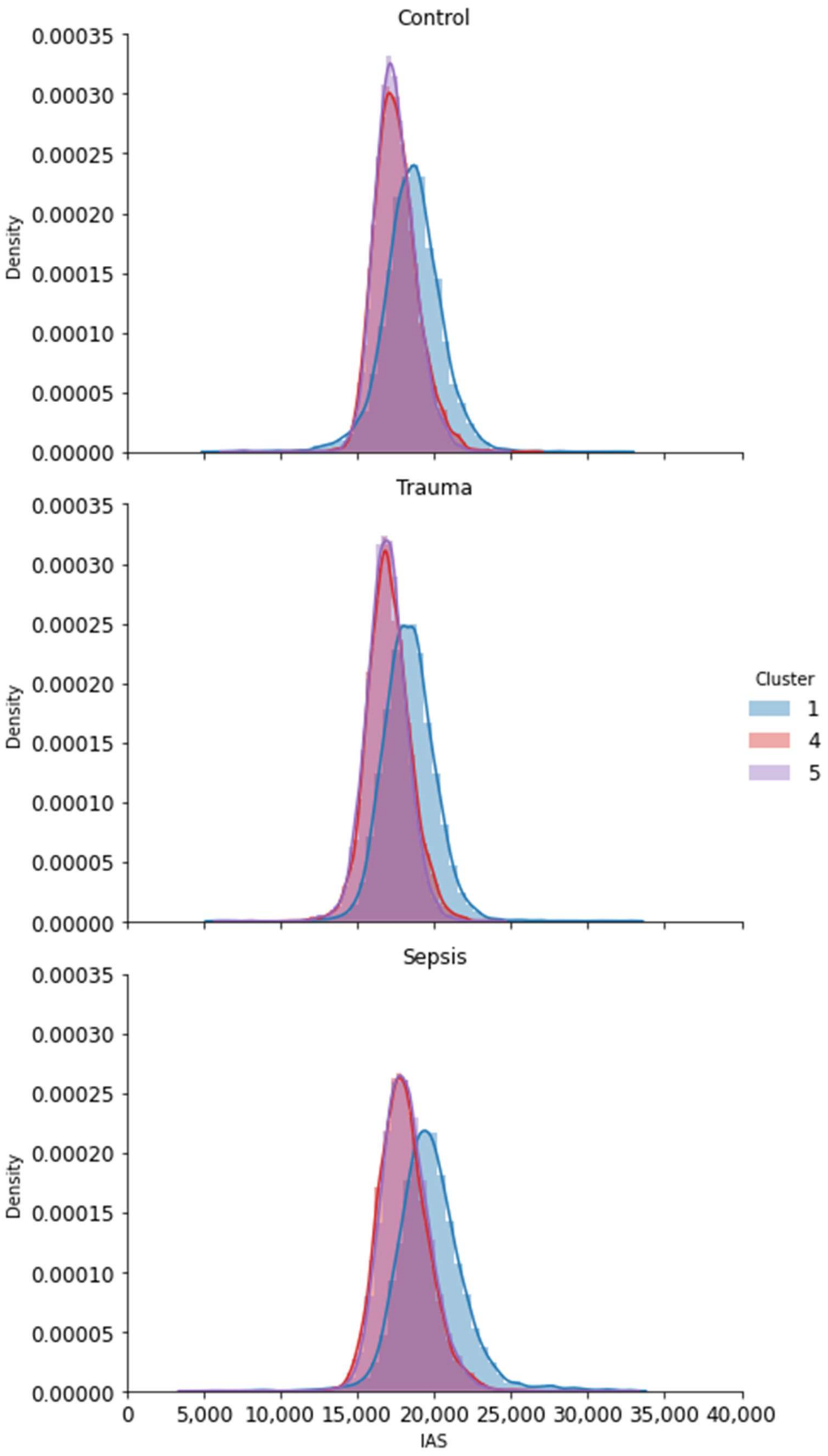

Figure S10. Density plots of IAS distributions, colorized according to cluster from K-means clustering. Although cluster 1 is shifted to the right, there is no clear distinction between the three clusters

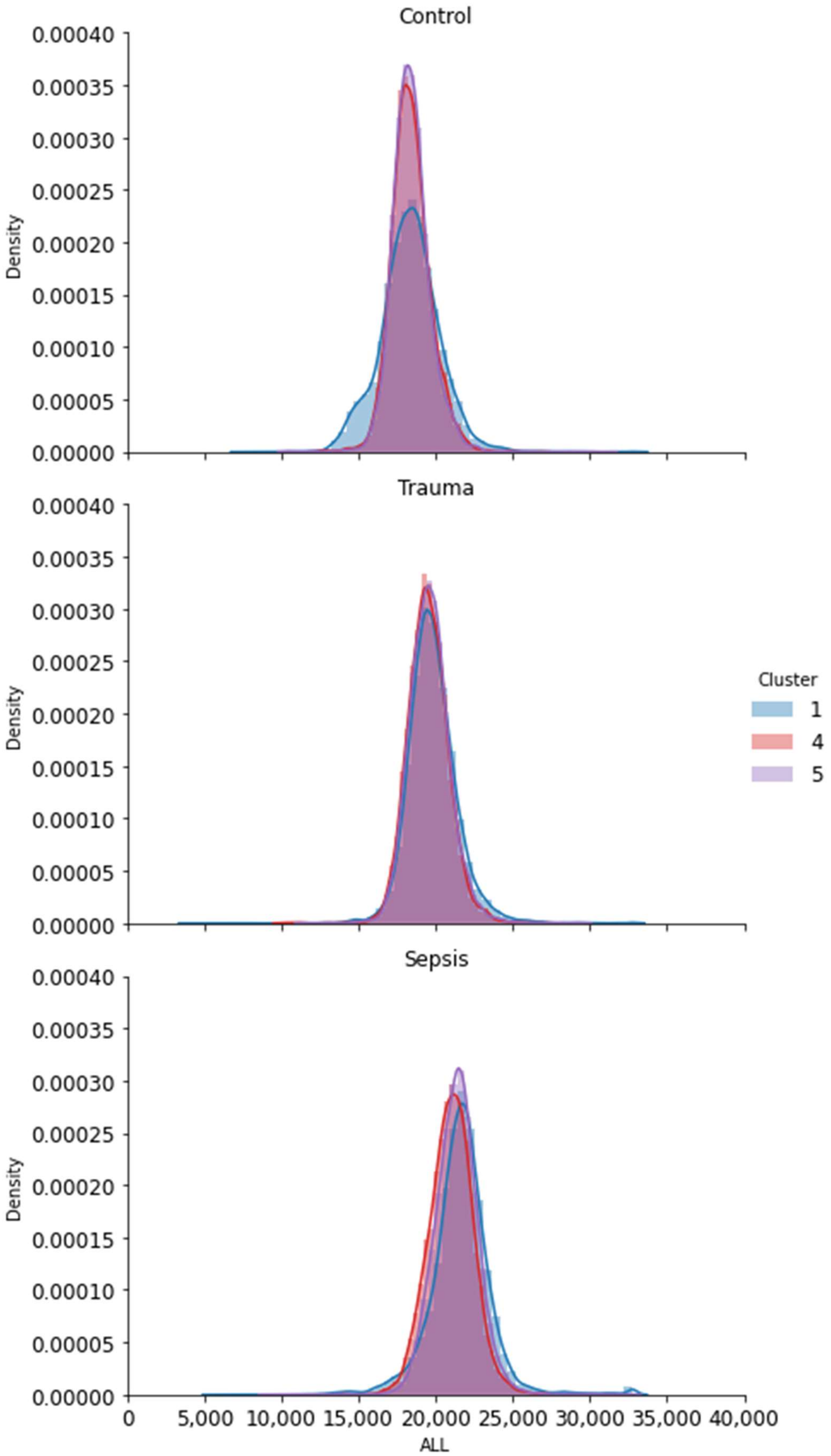

Figure S11. Density plots of ALL distributions, colorized according to cluster from K-means clustering. There is no clear distinction between the three clusters
