# Supplementary material for: Describing Characteristics and Differences of Neutrophils in Sepsis, Trauma, and Control Patients in Routinely Measured Hematology Data
Source: Biomedicines. 2022 Mar 9;10(3):633. doi: 10.3390/biomedicines10030633 (PMC8945568; doi:10.3390/biomedicines10030633)
Supplement: Supplementary file 1 [file biomedicines-10-00633-s001.zip › Supplementary Material 1.pdf]

Supplementary Material 1

Considering the findings as shown in Supplementary Figure S1a and b, the K-means clustering was performed using 5 clusters. The number was chosen as, besides k = 2, the silhouette score was highest for k = 5. With regards to choosing k = 5 over k = 2, Supplementary Figure S1b shows that a higher number of clusters is preferred, because the absolute KM-inertia is lower for more cluster, which is ideally as low as possible. However, the figure also shows that for K > 5, the additional reduction in the absolute score is limited (also known as the 'elbow' of the graph).

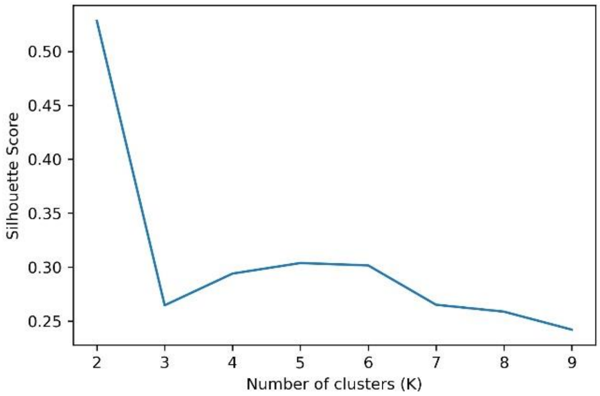

(**a**)

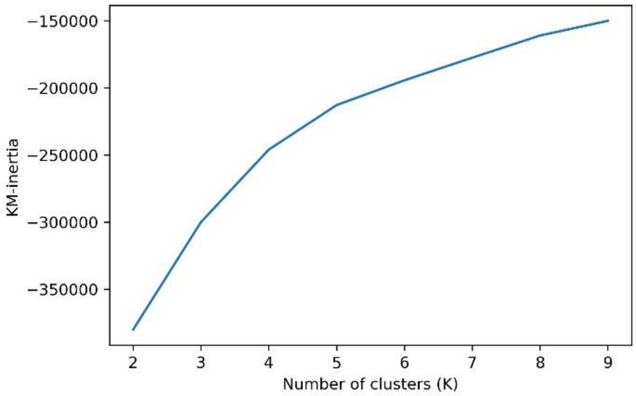

(**b**)

Figure S1. Scores with a different number of clusters for the K-means alghoritim. While there is a maximum at two clusters for the silhouette score, 5 clusters were used, as there is a decrease in information gain for the K-means inertia score (a) silhouette score, with a maximum at K=2, and then at K=5 (b) K-means inertia score for different values of K, showing a drop-off in gain at K=5.
