# Supplementary material for: Describing Characteristics and Differences of Neutrophils in Sepsis, Trauma, and Control Patients in Routinely Measured Hematology Data
Source: Biomedicines. 2022 Mar 9;10(3):633. doi: 10.3390/biomedicines10030633 (PMC8945568; doi:10.3390/biomedicines10030633)
Supplement: Supplementary file 1 [file biomedicines-10-00633-s001.zip › Supplementary Table 1.pdf]

Table S1

Table S1. Results of the Wilcoxon Rank Sum test between the three patient groups for the ALL variable

Comparison	$\|W\|$	ALL
Control vs Trauma	94.9	<0.001
Sepsis vs Trauma	136.7	<0.001
Sepsis vs Control	195.1	<0.001
